# Artificial intelligence in detecting temporomandibular joint osteoarthritis on orthopantomogram

**DOI:** 10.1038/s41598-021-89742-y

**Published:** 2021-05-13

**Authors:** Eunhye Choi, Donghyun Kim, Jeong-Yun Lee, Hee-Kyung Park

**Affiliations:** 1 Department of Oral Medicine and Oral Diagnosis, School of Dentistry and Dental Research Institute, Seoul National University, #101, Daehak-ro, Jongro-gu, Seoul, 03080 Korea; 2 Department of Advanced General Dentistry, Yonsei University College of Dentistry, Seoul, 03722 Korea; 3 Seoul Cheongchoon Dental Clinic, Seoul, 03086 Korea

**Keywords:** Diseases, Medical research

## Abstract

Orthopantomogram (OPG) is important for primary diagnosis of temporomandibular joint osteoarthritis (TMJOA), because of cost and the radiation associated with computed tomograms (CT). The aims of this study were to develop an artificial intelligence (AI) model and compare its TMJOA diagnostic performance from OPGs with that of an oromaxillofacial radiology (OMFR) expert. An AI model was developed using Karas’ ResNet model and trained to classify images into three categories: normal, indeterminate OA, and OA. This study included 1189 OPG images confirmed by cone-beam CT and evaluated the results by model (accuracy, precision, recall, and F1 score) and diagnostic performance (accuracy, sensitivity, and specificity). The model performance was unsatisfying when AI was developed with 3 categories. After the indeterminate OA images were reclassified as normal, OA, or omission, the AI diagnosed TMJOA in a similar manner to an expert and was in most accord with CBCT when the indeterminate OA category was omitted (accuracy: 0.78, sensitivity: 0.73, and specificity: 0.82). Our deep learning model showed a sensitivity equivalent to that of an expert, with a better balance between sensitivity and specificity, which implies that AI can play an important role in primary diagnosis of TMJOA from OPGs in most general practice clinics where OMFR experts or CT are not available.

## Introduction

Temporomandibular joint osteoarthritis (TMJOA) is an important subtype of temporomandibular disorders (TMDs) and may lead to substantial joint pain, dysfunction, dental malocclusion, and reduced health-related quality of life^1^. Osteoarthritis (OA) is a disease of joints caused by a series of degenerative processes including gradual loss of joint cartilage, remodeling and hardening of subchondral bone, and formation of osteoproliferative bodies^2^. TMJOA is confirmed by structural bony changes observed on radiographic examination. Because of the ability to show minute changes in the integrity of the cortical and subcortical bone of the TMJ, it is widely accepted that computed tomography (CT) is the reference standard for the diagnosis of TMJOA^3^. Cone beam CT (CBCT), which has the benefit of lower radiation exposure than conventional CT, is reportedly as accurate for the detection of TMJOA^4^. However, CBCT is not the first choice for TMJOA examination in the normal clinical setting yet because it still has a higher radiation dose and is costlier than plain radiographs. Normally, the orthopantomogram (OPG) is the most common examination method used for screening various lesions and conditions in the maxillofacial region, while it is less able to identify bony changes in the TMJ structure that are small in size and overlapped by other skull structures^5^. This makes OPG useful for screening examinations that experienced experts such as oromaxillofacial radiology (OMFR) or orofacial pain specialists read and then recommend, if necessary, an additional CBCT to confirm a diagnosis. In a situation where such experts are not available, a patient’s TMJOA can be overlooked or misread. To address this possibility, an AI algorithm was developed and trained to read TMJOA on OPGs based on CBCT results already confirmed by experts. Various studies have applied AI algorithms to read OPG for clinical conditions such as tooth segmentation^6^, age estimation^7^, third molar and inferior alveolar nerve detection^8^, cysts and tumors of the jaw^9^, osteoporosis^10^, and maxillary sinusitis^11^**.** However, there are few AI studies on TMJOA diagnosis; one study that used CBCT and one study based on OPG have been reported^12,13^.

This study aimed to investigate the clinical utility of an AI diagnostic tool developed for TMJOA diagnosis from OPG using deep learning that compared the AI read with that of an expert.

## Results

The results of the AI algorithm that was developed based on 3 categories, normal, indeterminate, and OA, are shown in Table 1. Because overall accuracy was not satisfying (Table 2), the model development process was modified and reevaluated because indeterminate TMJOA could have compromised AI training because of its vagueness on radiographic reading^14,15^. The AI was trained, validated, and tested again in 3 ways after modification. Indeterminate OA was considered as normal in Trial 1, OA in Trial 2, and omitted from the whole development process in Trial 3. The accuracy of the model performance was best in Trial 1 (0.80) followed by Trial 3 (0.78) and Trial 2 (0.73), but precision and recall were evenest in Trial 3 (Table 3). The recall value of TMJOA was 0. 51, which means that the model predicted TMJOA in patients with actual TMJOA about half the time in Trial 1. In screening tests, it is important to suspect the presence of the disease so that additional tests can be performed if necessary. For this reason, Trial 3 was chosen as a more suitable model in spite of the higher overall accuracy seen in Trial 1. Five-fold cross validation was performed on Trial 3. The average accuracy, precision, recall, and F 1 score were 0.76, 0.80, 0.71, and 0.75, respectively (Table 4).

**Table 1:** Clinical and demographic characteristics of the OPG dataset.

		Normal	Indeterminate	OA
Female	Number of joints	619	656	683
	Mean age	34.70	34.03	41.48
	95% CI	33.56–35.83	32.92–35.14	40.23–42.73
	SD	14.43	14.54	16.66
Male	Number of joints	181	123	116
	Mean age	31.86	28.20	32.88
	95% CI	29.78–33.94	26.11–30.28	29.88–35.88
	SD	14.27	11.78	16.50
Total	Number of joints	800	779	799
	Mean age	34.06	33.11	40.23
	95% CI	33.06–35.05	32.11–34.11	39.06–41.40
	SD	14.43	14.28	16.89

Indeterminate, indeterminate temporomandibular joint osteoarthritis; OA, temporomandibular joint osteoarthritis.

**Table 2:** Confusion matrix and model performance for the initial AI.

Confusion matrix				Model performance					
Actual	Predicted			Precision	Recall	Accuracy	Weighted average precision	Weighted average recall	F1 score
	Normal	Indeterminate	OA						
Normal	57	46	47	0.72	0.38	0.51	0.55	0.51	0.53
Indeterminate	14	53	83	0.44	0.35				
OA	8	22	120	0.48	0.80				

Indeterminate, indeterminate temporomandibular joint osteoarthritis; OA, temporomandibular joint osteoarthritis.

**Table 3:** Confusion matrix and model performance in each Trial.

Confusion matrix				Model performance					
	Actual	Predicted		Precision	Recall	Accuracy	Weighted average precision	Weighted average recall	F1 score
		Normal	OA						
Trial 1	Normal	283	17	0.80	0.94	0.80	0.81	0.80	0.80
	OA	72	78	0.82	0.52				
Trial 2	Normal	35	115	0.81	0.23	0.73	0.75	0.73	0.74
	OA	8	292	0.72	0.97				
Trial 3	Normal	119	26	0.78	0.82	0.78	0.78	0.78	0.78
	OA	34	93	0.78	0.73				

OA, temporomandibular joint osteoarthritis.

**Table 4:** Five-fold cross-validation in Trial 3.

Work	Precision	Recall	F1 score	Accuracy	AUC (95% CI)
1	0.82	0.59	0.69	0.74	0.83 (0.79–0.88)
2	0.80	0.71	0.75	0.77	0.86 (0.82–0.90)
3	0.83	0.76	0.79	0.81	0.87 (0.83–0.91)
4	0.75	0.76	0.75	0.75	0.83 (0.79–0.88)
5	0.77	0.74	0.76	0.76	0.84 (0.80–0.89)
Average	0.80	0.71	0.75	0.76	0.85 (0.81–0.89)

**Figure 1:**
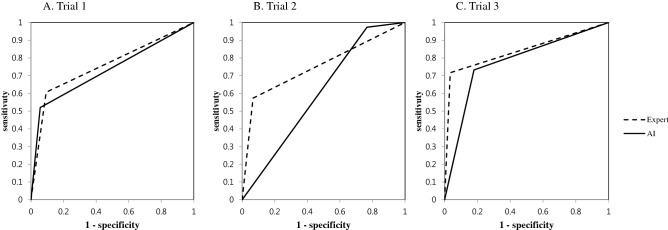
Comparison of the sensitivities and specificities in Trials 1, 2, and 3.

The diagnostic performance of the AI and expert and agreement between their OPG read and CBCT reads is shown in Table 5. The comparison of sensitivities and specificities in Trials 1, 2, and 3 are shown in Fig. 1. The AI in Trial 1 (0.80) and the expert in Trial 3 (0.85) were the most accurate, respectively. However, taking sensitivity and specificity into consideration collectively, it can be said that Trial 3 was the most accurate (0.85 for the expert and 0.78 for the AI). Cohen’s kappa was highest as well in Trial 3 and showed a substantial level of agreement for the expert (0.69) and moderate agreement for the AI (0.56). In all 3 trials, the expert read more accurately than the AI. However, the result of McNemar’s test showed that AI reads were more in accord with CBCT (*p* = 0.366) in Trial 3 where TMJOA was diagnosed dichotomously.

**Table 5:** Diagnostic performance and level of agreement in each Trial.

		Diagnostic performance			Cohen’s kappa	Kappa index	McNemar’s test
		Accuracy	Sensitivity	Specificity			
Trial 1	Expert	0.81	0.61	0.91	0.54	Moderate	.001
	AI	0.80	0.52	0.94	0.51	Moderate	.000
Trial 2	Expert	0.69	0.57	0.93	0.42	Moderate	.000
	AI	0.73	0.97	0.23	0.25	Fair	.000
Trial 3	Expert	0.85	0.72	0.97	0.69	Substantial	.000
	AI	0.78	0.73	0.82	0.56	Moderate	.366

AI, artificial intelligence.

## Discussion

OPG is the most widely used plain radiograph method for the primary diagnosis of TMJOA. However, OMFR experts or CBCT are not always available in most general practice clinics and screening for TMJOA is easily compromised. As reported previously, age, pain, and TMJ noise were not correlated with TMJOA on CBCT, while a high incidence rate for OA changes in TMD patients was observed (27.3%)^16^. It was also reported that 24% of patients who did not show significant condyle bone changes on OPG had degenerative bone changes on CBCT^17^. Moreover, it is also very well known that the accuracy, sensitivity, and specificity of OPG is not good for the diagnosis of TMJOA, even when an expert OMFR radiologist reads OPG images. OPG showed lower sensitivity (0.26) and higher specificity (0.99) compared to CT in TMJOA patients^14^. The diagnostic accuracy of OPG for detecting cortical erosion of the mandibular condyle is less (0.55–0.64) compared to CBCT (0.77–0.95)^18^. Because the bone tissue must be demineralized sufficiently before becoming noticeable on OPG, which usually takes more than 6 months, the mandibular condyle may appear normal on OPG in the early stages of TMJOA^19,20^. According to the position paper from the American Academy of Oral and Maxillofacial Radiology (AAOMR), OPG is only useful for diagnosing advanced TMJOA due to its low sensitivity^21^. Image distortion and overlap on OPG are always concerns as well^22^. However, considering the prominent role of OPG in primary examinations, any supplementary diagnostic tool to screen for TMJOA on OPG would be very helpful in clinics.

The AI algorithm developed in this study showed sufficient sensitivity compared to OMFS experts for the primary diagnosis of TMJOA on OPG. The diagnostic performance (accuracy, 0.51, and F1 score, 0.53) was not satisfying in the initial trial when AI was trained with 3 categories of TMJOA: normal, indeterminate TMJOA, and TMJOA. The Cohen’s kappa value for the AI diagnosis on OPG was 0.27, which was less than that of the expert, 0.38, while both represent fair agreement. This implies that it is still difficult to distinguish subtle changes in TMJOA on OPG even for an expert. Besides, multi-label image classification is more challenging than single-label classification^23^ as shown in a previous study that reported low performance (mean accuracy, 0.51) for AI classification of lower third molar development into multiple stages for age estimations^7^. Based on this initial result, classification of images was done in different ways.

When indeterminate OA was taken as normal in Trial 1, the AI model performance was best, and showed the highest accuracy, precision, recall, and F1 score (0.80, 0.81, 0.80, and 0.80, respectively). But, Trial 1’s sensitivity in diagnostic performance was lowest (0.52). In Trial 2 when indeterminate OA was defined as OA, the model performance (accuracy, 0.73, precision, 0.75, recall, 0.73, and F1 score, 0.74) and specificity (0.23) in diagnostic performance were lowest, while sensitivity was highest (0.97). In Trial 3 when indeterminate OA was omitted from the development process, the model performance (accuracy, 0.78, precision, 0.78, recall, 0.78, and F1 score, 0.78) came close to that in Trial 1, while Trial 3’s sensitivity (0.73) and specificity (0.82) were good and more balanced in terms of diagnostic performance. Based on this result, AI performed best for TMJOA diagnosis on OPG when indeterminate OA was omitted during training and verification. The pilot study showed similar accuracy (0.77–0.84) with various AI models when indeterminate OA was omitted^13^.

Indeterminate OA was recategorized as normal in previous studies^12,14^ as in Trial 1 of this study. The sensitivity of the AI (0.52) and expert (0.61) was higher and specificity was lower (0.94 for AI and 0.91 for expert) than those of experts in the previous study (sensitivity, 0.26 and specificity, 0.99)^14^. The model performance (accuracy, 0.86 and F1 score, 0.84) in a recent study^12^ was better than in Trial 1 of this study (accuracy, 0.80 and F1 score, 0.80) but the materials for AI development were CBCT sagittal images in that previous study. CBCT images have higher detail and fewer artifacts at the anatomical boundaries of the ROIs and background than OPGs^24^. The superiority of CBCT to OPG in the performance of a DCNN model has already been reported^25^.

Consequently, the AI algorithm in Trial 3 is the most appropriate for TMJOA diagnosis on OPG, and showed the best balance between sensitivity and specificity among our 3 trials and equivalent diagnostic performance to the expert. Moreover, no statistical difference was observed between the AI diagnosis on OPGs and the expert diagnosis on CBCT only in Trial 3 (McNemar’s test, *p* = 0.366), which must be because of a better balance between sensitivity and specificity. This implies that the AI model is more likely to accurately diagnose TMJOA on OPG in accord with expert diagnosis using CBCT when indeterminate TMJOA is excluded from AI training. When the AI in Trial 3 read 115 untested OPG images of indeterminate TMJOA (data not shown), AI was more likely to read indeterminate TMJOA as TMJOA (41 OPGs, 35.7%) than the expert (23 OPGs, 20.0%). Indeterminate TMJOA may be considered a normal variation, aging, physiologic remodeling, or a precursor to TMJOA^15^, which means it can be diagnosed as normal and OA at the same time. Because the benefit of high sensitivity may exceed loss of low specificity in the diagnosis of TMJOA where early detection is important, it would be clinically more beneficial for a TMJOA screening tool to read indeterminate OA as OA. In a randomized controlled study, osseous condylar changes in adolescents/young adults with early‐stage TMJOA showed repair and even regeneration after conservative and splint therapy^26^, which may emphasize the need for early detection and management.

Additional statistics were performed to explain Trial 3’s model prediction—which included factors correlated with the expert or AI’s diagnosis of TMJOA based on the OMFR expert’s CBCT reads. Location and types of degenerative bone changes based on CBCT reads by the expert were evaluated by Mann–Whitney test. The expert’s diagnosis of TMJOA was correlated with surface erosion (*p* = 0.001) and generalized sclerosis (*p* = 0.040), while AI’s diagnosis was correlated with surface irregularity (*p* = 0.033). In this study, AI was trained to learn the whole image with the extracted ROIs, and as a result, it might be said that image overall appearance could influence TMJOA diagnosis rather than specific OA changes. This may explain why AI was closer to CBCT than the expert in diagnosing TMJOA with OPGs.

AI is an up-and-coming method for use in radiology, where a large amount of homogeneous image data are available, and this includes AI’s strengths in segmenting, detecting, or classifying organs and lesions. Despite the lack of quality and number of studies, AI has been constantly shown to be equivalent to health-care professionals with respect to diagnostic performance^27^. On the other hand, AI is more likely to show better results when it is trained with an ROI image than with a whole image^28^. However, ROIs are extracted manually in most studies, which can compromise AI’s practical usability. In this study, an AI algorithm was designed to extract ROIs from OPG images based on an object detection technique. The average precision of mandibular condyle detection was reported to be 99.4% on right side and 100% on left side in a previous study^13^. The ROI extracted automatically for this study covered the mandibular condyle, and the articular fossa and eminence, which is very important because TMJOA is not defined only by bony changes in the condyle. It is obvious that OA changes occur in other structures of the TMJ such as the fossa and eminence^29^, so that the ROI for AI diagnosis should cover all structures of the joint, rather than just the condyle. But, most TMJOA studies usually focus on the condyle and only one AI study based on TMJ CBCT also used the mandibular condyle as extracted from sagittal sections^12^.

This study has several limitations. First, the absolute size of the training dataset was rather small. The use of a larger dataset for training and/or external validation may render different findings. In order to overcome the limitation of a small size dataset, data augmentation by image modification and a transfer learning technique with fine-tuning based on an imagenet database (http://image-net.org) were used. It has been reported that the accuracy of transfer learning increases to some extent for medical images rather than general objects^30^. Although AI did not exceed an expert with respect to accuracy in this study, this may improve in further studies with larger datasets, learning OPG and CBCT images together, elaborating deep learning models using landmarks, and an ensemble composed of multiple AI models. Second, it is well known that an external test dataset drawn from multiple medical centers secures high AI performance for reproducibility in general use. However, this study was based on an internal dataset, but, recently, there is no noticeable difference in performance between OPG devices from various manufacturers. Last, deep learning algorithms have inherent uncertainty^31^. Compared to machine learning methods based on handcrafted features, the trained deep learning model is like a “black-box” so the results are not explainable^32^.

## Conclusions

Collectively, an AI may read OPGs to diagnose TMJOA as well as OMFR experts can with respect to sensitivity and has greater accordance with CBCT interpretations than do OMFR experts, which implies that AI can play an important role in diagnosing TMJOA primarily from OPGs in most general practice clinics, where OMFR experts or CBCT are not available.

## Methods

### Materials

The written documentation of informed consent was waived and approved by the decision of the Institutional Review Board of School of Dentistry, Seoul National University (S-D20200004) and ethics committee approval for the study in the same institute was also obtained. All methods were performed in accordance with relevant guidelines and regulations. Radiographic images of the patients who visited the orofacial pain clinic of Seoul National University Dental Hospital who reported TMD-related symptoms and had an OPG (Orthopantomograph OP, 100D, Instumentarium Corporation, Tuusula, Finland) and TMJ CBCT (SOMATOM Sensation 10, Siemens, Erlangen, Germany) which were read by OMFR specialists from January, 2015 to October, 2019 were reviewed. Records of patients under 18 years of age or with a history of orthognathic surgery, macro trauma, and systemic diseases that could cause joint deformity or with a temporal difference of more than 3 months between OPG and CBCT imaging were excluded. The AI algorithm was trained, validated, and tested with 1189 OPGs, all of which had been confirmed by additional CBCT examination, selected randomly and classified by an orofacial pain specialist in terms of image analysis criteria for the diagnosis of temporomandibular disorder (research diagnostic criteria for TMD^14^; diagnostic criteria for TMD^15^: no TMJOA (normal), indeterminate for TMJOA (indeterminate), and TMJOA (OA). Among 2378 joints, 800 were diagnosed as normal, 779 as indeterminate, and 799 as OA based on the CBCT reads (Table 1).

### AI model development

**Figure 2:**
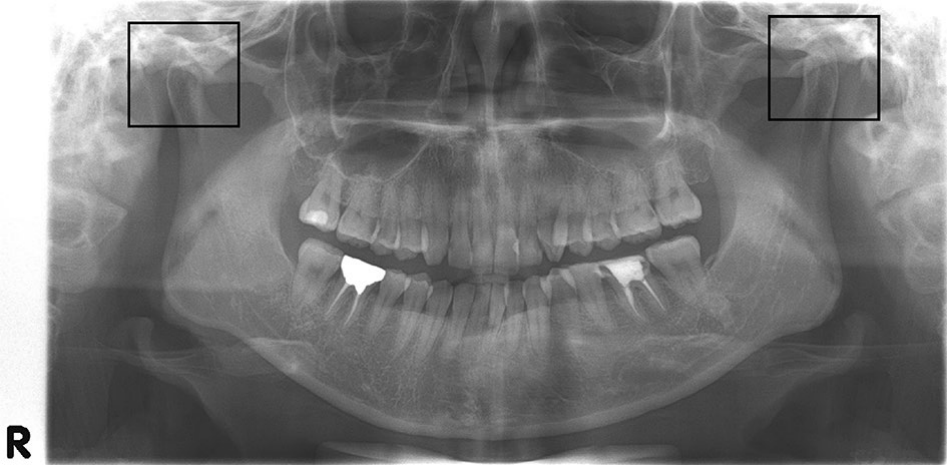
Result of ROI extraction, 300 × 300 pixels.

**Figure 3:**
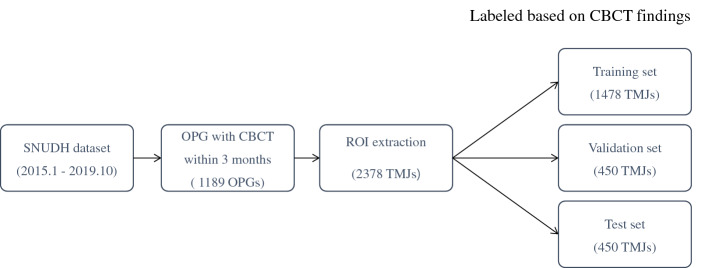
Clinical datasets used for training, validation, and test.

First, an algorithm to extract regions of interest (ROI) including the mandibular condyle and surrounding structures from each OPG by object detection was developed (Fig. 2). The objective detection technique was based on Faster Regions with Convolutional Neural Networks (RCNN) using the Inception V3 model as the categorizing algorithm in which Region Proposal Network (RPN) and Image Classification Network (ICN) work simultaneously and make it faster. The RCNN used an algorithm called selective search to extract approximately 2000 areas where there were likely objects. This technique is called region proposals. For each region, 4096-dimensional feature vectors were derived using CNN for image classification (Inception ResNet V2 was used in this paper). The CNN model took a 227 × 227 color image as the input and derived the included characteristics through 5 convolutional layers and 2 fully-connected layers. Therefore, region proposals must be warped to a size of 227 × 227 before putting them into the CNN. Then, a support vector machine was used to predict the class associated with the feature vector. Finally, Bounding-box (BB) regression was performed to determine the location of the objects more accurately. In the next step, extracted ROI images were classified as normal, indeterminate, and OA by means of the Keras’ ResNet model based on a Convolutional Neural Network (CNN). The ROI images of 2378 joints were divided randomly into training (1478 images), validation (450 images), and test sets (450 images), with which the AI algorithm was developed using Keras’ ResNet model. The test set consisted of 150 normal, 150 indeterminate, and 150 OA images (Fig. 3). Data augmentation was done by image rotation ± 5 degrees, image shift ± 10%, brightness ± 10%, and contrast ± 10% to compensate for the disadvantage of the small number of the data points to increase model robustness. Training and validation were repeated 35,000 times (700 epochs) with augmented data. The learning rate of the model was 1.0 × 10^–6^ and an Adam optimizer was used. After 700 training epochs, the validation loss of the model decreased from 12.2 to 0.1. In order to find the most suitable model for screening TMJOA, Indeterminate OA was treated as follows during AI model development.

1. Initial trial: Indeterminate OA was treated independently.
2. Trial 1: Indeterminate OA was considered normal.
3. Trial 2: Indeterminate OA was considered TMJOA.
4. Trial 3: Indeterminate OA was omitted.

After selecting the optimal trial, five-fold cross validation was performed to evaluate model training, while avoiding overfitting or bias. The 1599 images consisting of 800 normal and 799 OA selected as the training dataset were randomly divided into five folds. Within each fold, the dataset was partitioned into independent training and validation sets, using an 80 to 20 percentage split. The selected validation set was a completely independent fold from the other training folds, and it was used to evaluate model training status during the training. After one model training step was completed, the other independent fold was used as a validation set and the previous validation set was reused, as part of the training set, to evaluate the model training.

### Model and statistical analysis

Accuracy, precision, recall, and F1 score were calculated for model performance. Accuracy is defined as the ratio of correct predictions. Precision is the ratio of true positives to true positives and false positives. Recall is the ratio of true positives to true positives and false negatives. Finally, the F1 score is a harmonic mean of precision and recall: (2 × precision × recall)/(precision + recall). Accuracy, specificity, and sensitivity were calculated for diagnostic performance, Cohen’s kappa was calculated to estimate the agreement of TMJOA diagnosis between OPG and CBCT reads, and McNemar’s test was done to evaluate the significance of difference. For evaluation of AI clinical usability, the results between OPG reads by the AI and the expert were compared. All *p* values < 0.05 were considered to be statistically significant. The Python programming language (v. 3.6), Tensorflow (v. 2.0) and a graphics card (GeForce GTX 2080) were used for analysis.

## Data Availability

The data that support the findings of this study are available from the authors upon reasonable request.
